# Effects of Dry Needling on Active Myofascial Trigger Points and Pain Intensity in Tension-Type Headache: A Randomized Controlled Study

**DOI:** 10.3390/jpm14040332

**Published:** 2024-03-22

**Authors:** Sofía Monti-Ballano, Sergio Márquez-Gonzalvo, María Orosia Lucha-López, Loreto Ferrández-Laliena, Lucía Vicente-Pina, Rocío Sánchez-Rodríguez, Héctor José Tricás-Vidal, José Miguel Tricás-Moreno

**Affiliations:** Unidad de Investigación en Fisioterapia, Spin off Centro Clínico OMT-E Fisioterapia SLP, Universidad de Zaragoza, Domingo Miral s/n, 50009 Zaragoza, Spain; smonti@unizar.es (S.M.-B.); lferrandez@unizar.es (L.F.-L.); l.vicente@unizar.es (L.V.-P.); 739468@unizar.es (R.S.-R.); hjtricas@unizar.es (H.J.T.-V.); jmtricas@unizar.es (J.M.T.-M.)

**Keywords:** tension-type headache, dry needling, active myofascial trigger points, neurologic manifestations, muscle tenderness, referred pain, clinical trial, headache intensity, perceived clinical change

## Abstract

Tension-type headache is the most prevalent type of headache and is commonly associated with myofascial pain syndrome and the presence of active myofascial trigger points. This randomized controlled trial aimed to assess the impact of dry needling on the total number of active trigger points, pain intensity, and perceived clinical change in tension-type headache subjects. Thirty-two subjects were randomly assigned to the control and dry needling groups. The presence of active trigger points in 15 head and neck muscles, the headache intensity, and the perceived clinical change were evaluated. A single dry needling technique was administered at each active trigger point across three sessions. Significant differences were observed in the post-treatment measures favouring the dry needling group, including reductions in the headache intensity scores (*p* = 0.034) and the total number of active trigger points (*p* = 0.039). Moreover, significant differences in the perception of clinical change were found between the control and treatment groups (*p* = 0.000). Dry needling demonstrated positive effects in reducing the number of active trigger points and improving the short-term headache intensity in tension-type headache patients. A single dry needling session applied in the cranio-cervical area resulted in a self-perceived improvement compared to the control subjects.

## 1. Introduction

Tension-type headache (TTH) is the most prevalent neurological disorder in developing countries. The disorder is characterized by recurrent episodes of mild to moderate pain with a duration of attacks ranging from 30 min to 24 h [1,2]. Headaches tend to be bilateral and are described as following a hat-band pattern in the forehead, back of the head and neck [1]. The latest World Health Organization atlas explains that between 50% and 75% of the adult population suffers from headaches [3]. The annual cost of headaches due to healthcare expenses and lost productivity amounts to almost EUR 173 billion in the 27 countries of the European Union [4], resulting in significant repercussions on academic performance and psychological well-being [5].

Despite its high prevalence, the causes and underlying factors of TTH are still under study. Its diagnosis is based on the clinical criteria published/proposed in the third edition of the International Classification of Headache Disorders (ICHD-3), which divides them into episodic TTH, infrequent and frequent, and chronic TTH [6]. Bendtsen et al. were the first to define a physio-pathological model based on the central sensitization process [7]. This model established that the pain modulation process was deregulated by the sensitization of supraspinal structures and a decrease in the antinociceptive activity. These processes are combined by the effect of peripheral sensitization due to the excessive excitation of myelinated (Aδ) fibers and unmyelinated (C) afferents from peripheral nociceptors of the myofascial tissue [8]. A recent manuscript studying the trapezius muscle, as one of the muscles most frequently associated with myofascial pain in the neck and head region, and the cutaneous branches of spinal nerves that traverse it, established an interesting link between the entrapment neuropathy of the cutaneous branches of the dorsal rami of the spinal nerve traveling through the muscle and its fasciae and the myofascial pain evoked by the muscle [9]. The deep fascia of the trapezius muscle and cutaneous branches of spinal nerves are closely interconnected, allowing mechanical forces to be transmitted from the fascia to the nerves, potentially stretching and irritating them. This can lead to a neuropathic component of myofascial pain syndrome in the trapezius muscle, characterized by irritative neuritis of the cutaneous branches of spinal nerves [9]. This fact highlights the association between TTH and disfunction in the myofascial tissue of the cervical spine.

TTH patients respond more intensely to painful stimuli, and also become more sensitive to a wider range of stimuli, resulting in hypersensitivity [8]. Recent studies have correlated this peripheral sensitization with the presence of myofascial trigger points (MTrPs), which are hypersensitive nodules with a local and referred pain pattern. These MTrPs are activated because of precursor factors such as muscle fatigue, mechanical overload, or psychological stress. They produce a pathological depolarization of the motor plate during muscle contraction, triggering an “adenosine triphosphate energy crisis”. These factors, when present in the region of MTrPs, generate high levels of allogenic substances as well as a decrease in pH [1,10]. Furthermore, the referred pain has been reasoned to result from the convergence of different afferents on the same second-order nociceptor [11]. In the specific case of TTH, the cervical and trigeminal afferents converge in the caudal trigeminal nucleus; specifically, this is in the C1–C3 root muscles and trigeminal nerves of V1 (optic) and V3 (mandibular) [11,12]. Given that nociceptive somatic afferents from the upper cervical muscles and the trigeminal nerve [13], especially the V1 (optic) and V3 (mandibular) nerves, meet on the same relay neurons, it is possible that supra-spinal structures integrate the signals in an incorrect way [11]. This may result in the localization of pain in structures distant from the actual site of the painful stimulus, a phenomenon known as muscle referred pain [11]. Thus, distinct spinal trigeminal neurons processing nociceptive information receive convergent afferent input from multiple peri cranial muscles, which may occur at the primary afferent level through the collaterals of nociceptors innervating peri cranial muscle compartments [14]. Thus, referred pain in the innervated areas of V1 (optic) and V3 (mandibular) triggered by nociceptive somatic afferents from the C1–C3 root muscles may contribute to TTH. It has been shown that blocking one of these inputs can normalize the central trigeminal activity, which may explain the beneficial effect of extracranial therapeutic manipulations in primary headaches such as TTH [14].

The definition of suprathreshold stimulus intensities has helped to establish the stimulus–response functions of pressure pain in patients with TTH in MTrP placement [1]. Consequently, the high prevalence of active MTrPs (AMTrPs) in the head, neck and shoulder muscles has been associated with generalized pressure hypersensitivity in TTH patients. Research has described AMTrPs related to headaches in the suboccipital [15], upper trapezius [16], sternocleidomastoid [16], obliquus capitis superior of the head [17], temporal [18], levator scapulae [19], masseter and splenius capitis [10]. Nevertheless, the relationship between local or referred pain in the genesis of TTH in all these areas has not yet been proven. Travell and Simons also described the relationships among the referred pain in the TTH pain area and the splenius cervicis, semispinalis, obliquus capitis inferior, occipitofrontalis posterior and anterior, and musculature of the zygomaticus major [20]. However, no previous studies have focused on the analysis of this musculature.

The treatment of TTH is highly variable and includes both non-pharmacological and pharmacological interventions [1]. Pharmacological treatment can be divided into acute and preventive, both of which remain a non-specific disease approach [2]. The treatment of acute TTH episodes using multiple oral non-steroidal anti-inflammatory drugs (NSAIDs) remains the first treatment option, recommended by the European Federation of Neurological Societies Task Force (EFNS-TF). Ibuprofen and ketoprofen, combined with caffeine, have shown the greatest efficacy in the treatment of TTH; this is compared to paracetamol or aspirin, with minimal alterations in the previous safety profile [21]. The preventive treatment of episodes is only recommended for frequent episodic and chronic TTH patients [1,2]. This preventive treatment includes first-line drugs such as tricyclic antidepressants (TCAs), including amitriptyline, and is supported by the highest level of evidence; however, it is also associated with a high frequency of adverse events [22]. Alternative antidepressants are supported by lower levels of evidence for TTH prevention and are reserved for patients that have reported severe adverse events or poor initial efficacy [1].

Extensive studies in the literature have studied the potential effectiveness of non-pharmacological treatments in TTH. Manual therapy targeting the inhibition of AMTrPs can be effective in reducing headache symptoms and the intensity, duration, and frequency of episodes in TTH [2,10,23,24]. Physiotherapy interventions, such as transcutaneous electrical nerve stimulation (TENS) therapy and neuromodulation, may also be beneficial in pain modulation [2,25]. Psychological treatments for TTH include EMG biofeedback to learn to recognize and control muscle tension, progressive relaxation training to manage tension during daily activities, and cognitive behavioural therapy to identify and change the stress-inducing factors that exacerbate headaches [26,27,28,29,30].

The only non-pharmacological therapy recommended by the National Institution for Health and Care Excellence (NICE) for TTH is invasive needle physiotherapy [31]. Dry needling (DN) is an invasive treatment used for reducing the pain associated with AMTrPs [32]. This technique has been found to provide improvements as an acute medication for headaches [30,33]. But there are few studies that have verified the effectiveness of DN in TTH [34,35]. Most researchers highlight the challenge of quantifying changes in pain intensity, as subjective pain scales are typically used to assess patient improvement [32]. Previous studies have not evaluated the deactivation of AMTrPs in TTH. Furthermore, even though existing studies have established the relationship between muscles exhibiting referred pain in TTH pain areas, to our knowledge, no studies have conducted an extensive evaluation of all muscles related to TTH. Therefore, the aim of this study was to evaluate the effect of DN on all the AMTrPs present in muscles that contribute to headaches, as well as its effect on pain intensity and perceived clinical change in subjects with TTH.

## 2. Materials and Methods

### 2.1. Study Design

This study was part of a larger randomized controlled trial that was conducted under single-blind conditions. The randomization process was carried out using the web application www.random.org, accessed on 1 October 2023. The total number of required subjects was entered, and the numbers were randomly assigned to either the control group (left column) or the experimental group (right column)

Following the initial assessment, an identification number (ID) was assigned to each subject.

The research was submitted to and approved by the Ethics Committee of Clinical Research of Aragon (CEICA) (protocol code PI23-418/date of approval: 18 October 2023). The CEICA issued a favourable assessment, attesting that the project complies with the legal requirements and ethical principles applicable.

The methods followed adhered to the ethical principles outlined in the latest version of the Declaration of Helsinki published by the World Medical Association. In addition, informed consent was obtained from all subjects involved in the study. It was also registered and approved in ClinicalTrials.gov, with the following ID: NCT06108180.

Furthermore, the study was conducted at a private physiotherapy clinic known as Centro Clínico OMT-E. This research received no external funding.

### 2.2. Sample

For the calculation of the sample size, we used the data published in a previous study by Berggreen et al. that included subjects with similar characteristics who had received a manual therapy approach for the treatment of AMTrPs [36]. This study obtained a final total number of AMTrPs in the treatment group of 12.6 ± 14.3 and a total number of AMTrPs in the control group of 42.1 ± 10.4. The sample size was calculated using GPower software, version 3.1.9 (https://www.psychologie.hhu.de/arbeitsgruppen/allgemeine-psychologie-und-arbeitspsychologie/gpower, accessed on 1 October 2023), with the selection of a *t*-tests family, two independent means, bilateral contrast with a risk of α = 0.05, a power of 0.80, and an allocation ratio of 1 subject per group. A minimum of 5 subjects per group were obtained.

**Figure 1:**
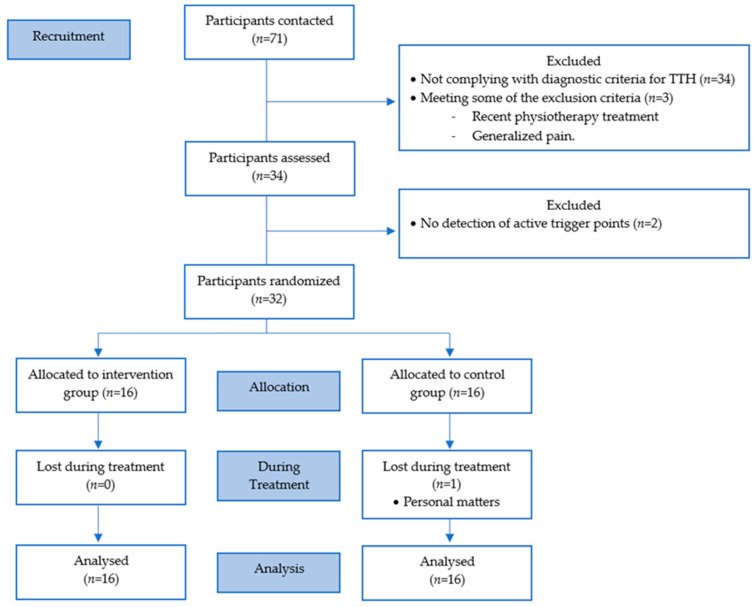
CONSORT (Consolidated Standards of Reporting Trial) flow diagram.

To improve the representativeness of the sample, 16 subjects per group were recruited (Figure 1).

The participant recruitment involved distributing posters on university campuses, utilizing social media platforms, and collaborating with physiotherapy clinics.

The inclusion criteria for participants were as follows: Patients were under the supervision of a neurology specialist. The diagnosis of TTH was based on the diagnostic criteria outlined in the International Classification of Headache Disorders, third edition of 2018 [6], for TTH. Participants exhibited the characteristic features of TTH, including bilateral pain, a pressing or tightening sensation, mild to moderate pain intensity, and no worsening of symptoms during physical activity. Additionally, participants were required to report no more than one of the following symptoms: photophobia, phonophobia, or mild nausea, without moderate or severe nausea or vomiting, as stipulated by the ICHD-III criteria. Additionally, participants were required to have at least one AMTrP in the muscles known to refer pain to the head, consistent with the diagnostic criteria and pain patterns described by Travell and Simons [37].

The exclusion criteria included significant trauma in the cervical area and/or recent surgery, pregnancy, a diagnosis of fibromyalgia, rheumatological, hormonal, or neurological disorders, chronic orthopaedic issues such as scoliosis, impingement syndrome, or acromion type 2. Participants were also excluded if they had a severe psychiatric condition, were unable to complete the form in Spanish, or had a pacemaker (due to the use of equipment with magnetic sensors). Additionally, individuals who had undergone physiotherapy treatment for headaches in the last month were not included.

### 2.3. Measurements

Clinical history: Patients were asked about their sex, age (years), and the frequency of headaches (days/month).

In relation to headache medication, participants were asked whether they were taking any type of the following 6 groups of medications: anti-inflammatories, analgesics, muscle relaxants, triptans, antidepressants, and/or anxiolytics. If the response was yes, they were asked to provide specific information about the medication (name and milligrams) and the number of doses they had taken per month.

#### 2.3.1. Pain Intensity

Pain intensity was evaluated with the visual analogue scale (VAS), which is a widely used tool for assessing pain intensity due to its strong psychometric properties, as well as its high reliability and validity [38]. The VAS was measured using a non-numbered vertical continuous line of 100 mm, where subjects had to mark their pain intensity with a horizontal line. The lower limit was the absence of pain, and the upper limit was the worst pain imaginable by a person. The evaluator subsequently measured the pain in millimetres.

#### 2.3.2. MTrP Examination

All muscles known to refer pain to the cranial or facial regions, which are typically associated with headaches according to Travell and Simons, were included for assessment. These muscles, examined bilaterally, encompassed the upper trapezius, splenius capitis and cervicis, semispinalis, rectus capitis posterior major, obliquus capitis superior and inferior, occipitofrontalis posterior and anterior, temporalis, masseter, clavicular and sternal head of sternocleidomastoid, and zygomaticus major. Additionally, the levator scapulae was examined, given prior findings suggesting the presence of AMTrPs in this muscle among patients with TTH [19].

The identification of MTrPs in the aforementioned area was performed considering the existence of a painful tender spot within the muscle [37].

The diagnosis of AMTrPs among the previously identified MTrPs was conducted using algometry to mitigate the potential biases associated with manual pressure and a lack of quantitative pressure testing at the tender spot.

A digital algometer (Somedic AB Farsta, Sösdala, Sweden) was used for this purpose. The algometer’s pressure area was 1 cm^2^, and pressure was applied at a rate of 1 kg/cm^2^/s to each tender spot. Once the pressure reached the pressure pain threshold, it was sustained for 10 s to ascertain the activity status of the point.

An MTrP was considered active if the pain elicited during the pressure replication reproduced at least a portion of the TTH pain pattern typically reported by the patient. This ensured that the pain experienced was familiar and/or recognizable to the patient.

#### 2.3.3. Perceived Clinical Change

The Global Rating of Change Scale (GROC) was used to assess the perceived clinical change throughout the study. The GROC is a frequently used scale that was described over 25 years ago by Stratford et al. [39] and consists of a single question with 15 possible responses. These responses range from “much worse”, which assigns a score of −7 points, through “same as before”, with a score of 0 points, to “much better”, assigning a score of +7 points. This scale has shown good reliability and sensitivity in neck disorders [40].

### 2.4. Intervention

The total number of AMTrPs present in each subject was evenly distributed among the three treatment sessions. If the subject had fewer than three AMTrPs, a new assessment was performed in the corresponding treatment session. No AMTrP was treated more than once throughout the study.

The DN technique for each AMTrP involves a specific patient position and needle insertion adapted to the muscle. Prior skin disinfection was performed for all muscles to prevent any type of infection, and the same type of needle (0.32 mm × 40 mm) was used, except for the sternocleidomastoid, the zygomatic major, and the superficial musculature (occipitofrontal and temporal), for which a smaller needle (0.32 mm × 25 mm) was used.

**Figure 2:**
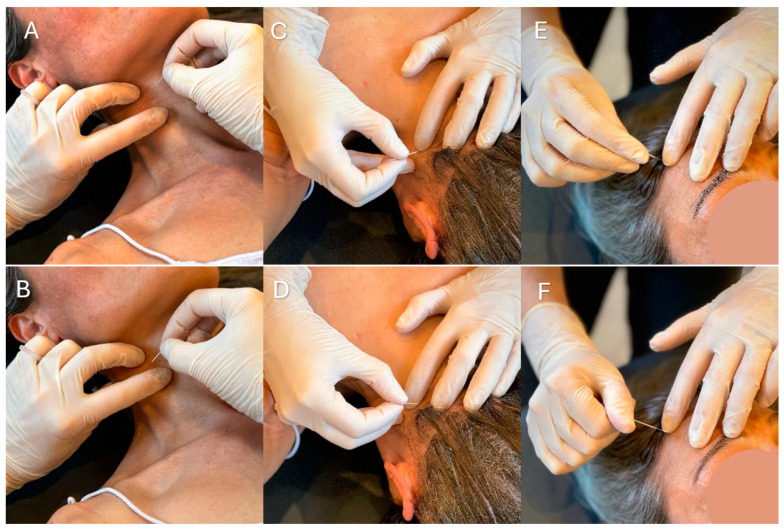
Demonstration of dry needling technique in different muscles. (**A**) Outward needling of the sternocleidomastoid muscle. (**B**) Inward needling of the sternocleidomastoid muscle. (**C**) Outward needling of the obliquus capitis inferior muscle. (**D**) Inward needling of the obliquus capitis inferior muscle. (**E**) Left rotation needling of the occipitofrontalis anterior muscle. (**F**) Right rotation needling of the occipitofrontalis anterior muscle.

The DN technique was applied directly to the AMTrP, aiming to reproduce all local twitch responses elicited by the AMTrP following the methodology described by Travell and Simons [20]. The techniques applied were as follows: in and out needling or needle winding in muscles with a large-diameter muscular belly and no nearby dangerous structures, and the bidirectional rotation technique was employed in muscles with a very flat muscular belly (such as the temporalis, occipitofrontalis posterior and anterior, and zygomaticus major muscles), and those with structures like blood vessels and nerves nearby (rectus capitis posterior major, obliquus capitis superior and inferior) (Figure 2).

### 2.5. Statistical Analysis

Age, headache frequency, headache medication, VAS, and the total number of AMTrPs were described using the mean and the standard deviation. The normality of these quantitative variables was tested using the Shapiro–Wilk test. Sex was described, and the percentages were offered.

Differences in the total number of AMTrPs, VAS and GROC between groups were analyzed with Student’s *t*-test for independent samples or with the Mann–Whitney test. Differences in the total number of AMTrPs and VAS between the pre- and post-treatment periods were analyzed with Student’s *t*-test for repeated measures or with the Wilcoxon signed-rank test in each group (control and intervention).

Two general linear models of repeated measures were constructed to model the dependent variables VAS and total number of AMTrPs as functions of the treatment group (intervention or control) as the between-subjects factor, the time (pre- and post-treatment measures) as the within-subjects factor, and the difference in the headache medication used between the pre- and post-treatment measures as a covariable.

One univariate general linear model was constructed to model the dependent variable GROC as a function of the treatment group (intervention or control) and the difference in the headache medication used between the pre- and post-treatment measures as a covariable.

The statistical analysis was performed on an intention-to-treat basis with Little’s test of missing completely at random and the expectation maximization option.

The data were analyzed with IBM SPSS Statistics Software version 25.0 (IBM Corporation, Armonk, New York, NY, USA). The statistical significance was established at *p* value < 0.05.

## 3. Results

The descriptive and comparative study of the sample at baseline is presented in Table 1. A total of 32 individuals were examined, with a mean age of 39.09 ± 12.88. The sample consisted of 75% women and 25% men.

Table 2 shows that there were no significant differences between the control and intervention groups in the post-treatment measurement of the dependent variables VAS and the total number of AMTrPs. The use of headache medication was significantly lower in the intervention group (*p* = 0.009). The GROC scale showed a more favorable outcome in the intervention group, with statistical significance (*p* < 0.000).

There were no significant differences between the pre- and post-treatment measures in the control group in the VAS or the total number of AMTrPs. However, in the intervention group, the VAS significantly decreased (Wilcoxon signed-rank test; *p* = 0.008).

In the general linear model of repeated measures for the dependent variable VAS, the difference in the use of headache medication between pre- and post-treatment as a covariable and the group and time as factors, there were not any significant main interactions; however, the interaction between group and time almost reached significance (*p* = 0.061). The post hoc analysis (Table 3) showed that at post-treatment, the value of the VAS in the intervention group was significantly lower than in the control group (*p* < 0.034). This supports the result obtained in the repeated measures analysis and suggests that the changes in the use of headache medication did not moderate the effect of the treatment in the VAS.

In the general linear model of repeated measures for the dependent variable “total number of AMTrPs”, the difference in the use of headache medication between pre- and post-treatment as a covariable and the group and time as factors, the interaction between group and time was statistically significant (*p* = 0.034). The post hoc analysis (Table 4) showed that at post-treatment, the value of the total number of AMTrPs in the intervention group was significantly lower than in the pre-treatment measure (*p* = 0.039). This suggests that the decrease in the use of headache medication in the intervention group favored the effect of the treatment in the total number of AMTrPs.

The univariate general linear model constructed to model the dependent variable GROC as a function of the treatment group (intervention or control) and the difference in the use of headache medication between the pre- and post-treatment measures as a covariable showed a significant effect for group (*p* < 0.000). Thus, the changes in the use of headache medication did not moderate the effect of the treatment.

No adverse events were reported with the intervention. Participants commonly reported light post-needling soreness in the upper trapezius, masseter and sternocleidomastoid muscles as a side effect of the technique. In every case, this side effect normalized in the next 48 h without needing specific treatment.

## 4. Discussion

This randomized controlled trial aimed to evaluate the effect of DN on the total number of AMTrPs in all muscles contributing to headaches, as well as on the pain intensity and perceived clinical change in TTH subjects. While prior evidence exists regarding the effects of DN on headaches [30,33], there is a scarcity of studies exploring its impact specifically on TTH. None of these studies have addressed the variables examined in this study. The most recent review suggests that future studies that have an improved classification of headache types and include less common variables are needed [30]. Participants who received the intervention experienced a decrease in the total number of AMTrPs and a decrease in headache intensity, as evidenced by the post hoc analysis. Additionally, they reported a significant improvement in the perceived clinical outcomes.

The total number of AMTrPs in the intervention group decreased by 4.50 compared to the beginning of the study. This difference is statistically significant, considering the effect of medication. This improvement was also observed in previous studies with manual soft tissue treatment [36]. However, none of these studies have investigated the effect of DN in reducing AMTrPs in subjects with TTH. The decrease in AMTrPs could have a clinical implication for the patient, being associated with the improved function of the affected musculature and a reduction, or even disappearance, of the referred pain caused by those AMTrPs [41]. It has been found that deep DN into the AMTrP of the upper trapezius ameliorates intramuscular blood circulation and muscle stiffness in females referred to physical therapy clinics due to neck or shoulder pain and with an AMTrP in the right upper trapezius muscle [42]. This amelioration in muscle stiffness may be due to local twitch responses, which have been shown to change the length and tension of the muscle fibers [43]. Local twitch responses, elicited by the mechanical needling activation of the muscle fibers around the end plate, have also been shown to suppress spontaneous electrical activity in AMTrPs [44,45]. It has been hypothesized that the amelioration of blood circulation after DN is related to the release of vasoactive substances, such as the calcitonin gene-related peptide which, upon activation of Aδ- and C-fibers, generates vasodilatation in small vessels [46].

It has been found that AMTrPs have higher levels of pro-inflammatory cytokines such as interleukin-1β and tumor necrosis factor-α compared to latent MTrPs and areas in the muscle that are not classified as MTrPs. These findings suggest that AMTrPs are associated with an inflammatory and sensitizing milieu [47]. An interesting study in a rabbit model found that one session of DN in the biceps femoris with MTrP enhanced the beta-endorphin levels in the biceps femoris muscle containing the taut band and serum, and reduced substance P in the biceps femoris and the ipsilateral corresponding segment of the L2–L5 dorsal root ganglion [48]; this shows the potential beneficial effect of the performance of one DN session on the MTrP with an in-and-out needle movement, eliciting as many local twitch responses as possible, in the inflammatory and sensitizing milieu related to AMTrPs. However, five daily DN sessions for five consecutive days showed pro-inflammatory effects, with increased levels of tumor necrosis factor-α, cyclooxygenase-2, and hypoxic-responsive proteins, such as hypoxia-inducible factor-1α, vascular endothelial growth factor, and the inducible isoform of nitric oxide synthases [48].

A recent interesting histological study in a rodent model of MTrP discovered that there is an abundance of glycosaminoglycans around the contraction knots in the cranial muscle of rats [49]. In human muscles, it is conceivable that both the contraction knots and the accumulated glycosaminoglycans might play a role in affecting the nodular texture that is frequently detected upon the palpation of MTrP [49]. As glycosaminoglycans are highly hygroscopic molecules, they might play a role in the entrapment of the nociceptive substances in the MTrP. Thus, the mechanical effect of dry needling may be pivotal in counteracting the aforementioned biochemical milieu, disrupting the chains of glycosaminoglycans and promoting the drainage of inflammatory cytokines.

The stimulation of both large, myelinated fibers (Aβ and Aδ fibers) and C-fibers produced by DN can contribute to pain relief in AMTrPs. The needle produces a mechanical stimulation of Aβ and Aδ fibers. The mechanical stimulation activates Aβ and Aδ fibers, which send afferent signals to the dorsolateral tracts of the spinal cord and activate the supraspinal regulation of pain [50]. However, it is thought that the mechanisms of pain regulation with DN are diverse and possibly confluent [51].

Moreover, it has been found that mechanical stimulation with the rotation of a needle in the connective tissue from the skin to the subcutaneous muscle has effects on connective tissue health and analgesic effects, with the analgesic effects being the most immediate and the healing of connective tissue being more delayed [52]. It has been shown that with small amounts of needle rotation, the tissues superficial to the muscle grasp the needle, pulling collagen fibers towards the needle; as a result, the bundles of collagen become straighter and more parallel. This also causes fibroblasts to move away from the needle and to change their shape, becoming larger and planer in contrast with the dendritic shape seen without needle rotation [52]. It has been hypothesized that the needle is grasped due to mechanical coupling between the needle and connective tissue; this transmits a mechanical signal to connective tissue cells via mechanotransduction, contributing to the healing process [53].

However, considering the type of DN performed, its target structure, and the AMTrPs within the muscle, we believe that the majority of the observed effect regarding the reduction in the total number of AMTrPs in our study can be attributed to the puncture’s impact on the hyperirritable spot of the muscle.

The effect of DN in reducing the intensity of headaches has been widely studied. We found a reduction of 8.69 mm in the VAS scale in the intervention group. In the latest studies on the effectiveness of DN on headache intensity, Gildir et al. [34] found a decrease of 38.5 mm in the VAS scale after six treatment sessions with DN, and Kamali et al. [35] found a decrease of 50 mm after three treatment sessions with DN. This difference may be due to disparities in the baseline headache intensity, as these authors found a mean of 45.5 mm and 87.5 mm, respectively, at the beginning of the study; this is compared to the mean of 19.31 mm found in our study. In another previous study on the use of DN for TTH, improvements were also observed in patients after the intervention, but the data are not comparable as the authors used a different intensity scale [54]. DN for AMTrPs has also been successful in reducing the pain intensity in other pathologies of the cervical and cranial region, such as myofascial temporomandibular disorders [55], neck pain [56] or cervicogenic headaches [57].

In our study, the subjects in the treatment group obtained a positive significant clinical change compared to the subjects in the control group. These DN results have also been observed in other types of patients, such as those with neck pain [58] or cervicogenic headaches [59]; however, in those studies, DN was not the only treatment technique used. Other physiotherapy techniques have also improved patients’ perception of their pain. Cabanillas et al. [60] achieved a significant improvement in 77.5% of patients treated with diacutaneous fibrolysis. In addition, Moraska et al. [61] achieved a significant improvement in 84.7% of subjects treated with friction massage on the AMTrPs. Understanding the patient’s perception of their clinical change and status is crucial for assessing the prognosis of the condition and customizing treatment approaches [62].

In this study, we did not register any serious adverse event apart from the usual post-needling soreness considered a side effect of the technique, with some authors considering this post-needling soreness to be a physiological consequence of the local twitch response [63]. A recent review on the effects of manual joint mobilization techniques, supervised physical activity, psychological treatment, acupuncture and patient education for patients with TTH found that no serious adverse events were identified for acupuncture, supporting the recommendation that this technique is used at the same level as manual joint mobilization or physical activity in TTH [64]. Another review and meta-analysis on DN for the treatment of TTH, cervicogenic, or migraine headaches found that in one of the studies, in 160 patients with TTH, five patients in the intervention group experienced pain and fear during the DN, but no other adverse events such as local infection or bleeding were registered [65].

### Limitations

This study provides preliminary evidence that DN significantly reduces the number of AMTrPs and headache intensity in the short term. However, the results cannot be generalized to the general population due to the limited sample size. Due to the lack of a placebo group, a potential placebo effect cannot be discarded. DN of the cervical muscles was performed without ultrasound guidance. Ultrasound guidance should be considered in future research to contrast the location and depth of the target muscles, as well as to accurately avoid the vascular and neural structures surrounding the cervical spine. Further studies with larger samples are needed to confirm these findings and determine the optimal treatment dose for DN in TTH.

Future studies are needed to explore the efficacy of DN compared to other physiotherapy techniques and assess its long-term effectiveness. Additionally, further studies should investigate the potential benefits of combining DN with other physiotherapy interventions, such as exercise therapy or manual therapy, for TTH.

Further research is recommended to investigate the efficacy of DN in comparison to other physiotherapy techniques, as well as the possibility of combining it with other therapies to achieve better outcomes.

## 5. Conclusions

DN has shown positive effects in reducing the number of AMTrPs that contribute to headache and improving the intensity of short-term headaches in patients with TTH.

A single session of DN applied in the cranio-cervical area of patients with TTH resulted in self-perceived improvement compared to the control subjects.

## Figures and Tables

**Table 1:** Descriptive and comparative study of the sample at pre-treatment.

	All the Sample	Control Group	Intervention Group	*p* Value
Age (years)	39.09 ± 12.68	41.44 ± 14.68	31.75 ± 10.75	0.308 **
Headache frequency (days/month)	13.44 ± 10.19	13.19 ± 11.55	13.69 ± 9.00	0.569 **
Headache medication (doses/month)	29.38 ± 53.73	39.81 ± 69.98	18.94 ± 28.21	0.227 **
VAS (mm)	19.31 ± 18.75	19.44 ± 19.07	19.19 ± 19.05	0.955 **
Total number of AMTrPs	15.53 ± 8.21	13.50 ± 8.02	17.56 ± 8.15	0.165 *

VAS: Visual Analogic Scale, AMTrPs: Active Myofascial Trigger Points. * Student’s *t*-test for independent samples. ** U de Mann–Whitney test.

**Table 2:** Descriptive and comparative study of the sample at post-treatment.

	Control Group	Intervention Group	*p* Value
Headache medication (doses/month)	46.63 ± 72.54	13.00 ± 25.03	0.009 **
VAS (mm)	27.93 ± 31.52	10.50 ± 16.79	0.160 **
Total number of AMTrPs	15.86 ± 7.83	13.25 ± 9.47	0.402 *
GROC	−0.38 ± 2.28	3.94 ± 2.08	0.000 **

VAS: Visual Analogic Scale, AMTrPs: Active Myofascial Trigger Points, GROC: Global Rating of Change. * Student’s *t*-test for independent samples. ** U de Mann–Whitney test.

**Table 3:** Post hoc analysis from the general linear model for the dependent variable VAS.

Dependent Variable: VAS (mm)	Mean Difference (Control Less Intervention Group)	Confidence Interval for the Difference	*p* Value
Post-treatment	23.45	1.89–45.01	0.034

VAS: Visual Analogic Scale.

**Table 4:** Post hoc analysis from the general linear model for the dependent variable AMTrPs.

Dependent Variable: Total Number of AMTrPs	Mean Difference (Pre Less Post-Treatment)	Confidence Interval for the Difference	*p* Value
Intervention	4.50	0.25–8.75	0.039

AMTrPs: Active Myofascial Trigger Points.

## Data Availability

The datasets analyzed during the current study are available from the corresponding author upon reasonable request. All data analyzed during this study are included in this published article.
